# Episodic apnea: gastroesophageal reflux associated with gastric organo-axial malrotation: a case report

**DOI:** 10.1186/s13256-022-03367-x

**Published:** 2022-04-05

**Authors:** Sena Turk, Sule Gokce, Feyza Umay Koc

**Affiliations:** Department of Pediatrics, Faculty of Medicine, Ege University Children’s Hospital, Bornova, Izmir, 35100 Turkey

**Keywords:** Gastroesophageal reflux, Apnea, Gastric organo-axial malrotation

## Abstract

**Background:**

Gastroesophageal reflux is a normal physiologic process occurring several times a day in healthy infants. On the other hand, symptoms such as failure to thrive, feeding or sleeping problems, chronic respiratory distress, persistent forceful vomiting, and choking may indicate reflux associated with underlying anatomic, neurological, or infectious abnormalities. Gastric malrotation is an extremely rare disorder in the pediatric population and one of the anatomic causes associated with severe reflux, which could lead to serious complications. In such cases, life-threatening symptoms overlapping with other diseases cause delayed diagnosis and treatment.

**Case presentation:**

We report a 2.5-month-old white girl diagnosed with gastric malrotation-related reflux, which caused inadequate weight gain, feeding difficulties, episodes of apnea with cyanosis, and choking after successive coughing, hence previously misdiagnosed as epilepsy and pertussis.

**Conclusion:**

Life-threatening symptoms in an infant with reflux suggest anatomic, neurological, or infectious conditions. Gastric malrotation is more common than generally thought and specifically looked for in young children with severe reflux symptoms, and should be diagnosed and treated as soon as possible.

## Background

Gastroesophageal reflux disease is a condition in which reflux of stomach contents into the esophagus produces troublesome symptoms that negatively affect quality of life [1]. Further evaluation is required to determine the underlying pathology in the presence of red flag symptoms such as failure to thrive, excessive irritability, feeding or sleeping problems, hematemesis, and respiratory distress [2]. Gastric malrotation, defined as torsion of the stomach around its short or long axis leading to dysfunction of the lower esophageal sphincter, is one of the anatomic causes of severe GER. It is an uncommon disease in the pediatric population. There have been only close to 600 cases of gastric malrotation in children published in the last century. Also, its clinical symptoms, including sudden onset of nonbilious emesis, epigastric pain, abdominal distention, and less commonly respiratory distress, cannot be interpreted easily [3]. All these features make it challenging to consider the diagnosis of gastric malrotation.

Infants with gastric malrotation have been thought to be at risk for reflux because they usually have a defective gastroesophageal junction during fetal development. In such cases, GER is thought to be responsible for respiratory symptoms such as cough, apnea, and recurrent lung infections, which could be life-threatening [4]. Although there are insufficient data to explain the association between reflux and episodic apnea, it is a fact that respiratory distress could be seen in children with severe reflux. It is hypothesized that pharyngeal penetration of reflux may activate local vagal stretch and trigger induction of apnea through pathways mediated by the brain stem [5].

Apnea is defined as cessation of breathing for more than 20 seconds or a shorter respiratory pause associated with bradycardia, cyanosis, and hypotonia. For an infant younger than 1 year, such an apneic episode associated with cyanosis, pallor, irregular breathing, marked change in tone, or altered level of responsiveness is defined as a brief resolved unexplained event (BRUE). Several conditions such as respiratory infections, seizures, sepsis, and gastroesophageal reflux can present with a brief apneic event [6]. Therefore, these cases should be evaluated diligently and even hospitalized to observe and search for another possible underlying condition.

Consequently, gastric malrotation-related reflux should be considered in the pediatric population with persistent respiratory problems. In such cases accompanied by severe complications of GER disease, the underlying causes should be diagnosed and treated quickly.

## Case presentation

A 2-month-20-day-old white girl, who was born at 38 weeks of gestational age with birth weight of 3 kg by cesarean section without complications, had been exclusively breastfeeding and was vaccinated on time. She had presented to the emergency department for the first time when she was 40 days old with complaints of episodes of choking, shortness of breath, and cyanosis after coughing. She experienced an apnea episode that lasted 35 seconds associated with cyanosis and an oxygen saturation level of 45%. Her muscle tone was normal, and there were no abnormal movements in her eyes or limbs. After the episode resolved, it was observed that the patient’s color returned to pink, the oxygen saturation level was 100%, and the physical examination findings were unremarkable. She was hospitalized with a prediagnosis of pertussis-like syndrome, and antibiotherapy was started as clarithromycin. Upon the increase in choking and apnea attacks with the negative result of the polymerase chain reaction (PCR) for *Bordetella pertussis*, clarithromycin was discontinued in 3 days. Epilepsy was another possible diagnosis because the patient had life-threatening repetitive hypoxemia, and symptoms resolved between the episodes. Therefore, phenobarbital was started at a dose of 5 mg/kg. However, due to lack of clinical improvement and absence of epileptic focus on electroencephalogram (EEG), antiepileptic therapy was discontinued. The patient was referred to our clinic for further medical investigation. She was hospitalized for evaluation of differential diagnoses such as gastroesophageal reflux, pertussis, aortic arch anomaly, infantile epileptic encephalopathy, and BRUE.

Physical examination revealed an afebrile, mildly dehydrated female infant in good general condition and interested in her environment. She had normal findings on auscultation with a respiratory rate of 30 breaths/min and oxygen saturation of 100% on room air. Cardiovascular examination revealed a systolic 1/6 murmur over the mesocardiac area and capillary refill time shorter than 2 s. Her body weight was 4.4 kg (3–10th percentile) at hospitalization, and she had poor weight gain, while her birth weight was 3 kg (25–50th percentile). The abdomen was mildly distended with normal active bowel sounds on auscultation without evidence of hepatosplenomegaly. The neurologic examination, including anterior fontanel and head circumference, was unremarkable. In the clinical follow-up, it was observed that she had episodes of apnea, cyanosis, and choking that began after successive coughing, lasted for 20–25 s, and up to 20 times a day. Episodes appeared approximately 15 min after feeding, sometimes accompanied by vomiting, showing cyanosis with oxygen saturation as low as 40%. Epilepsy was considered as another possible diagnosis because of repetitive periods and being asymptomatic between the episodes. However, the patient experienced apneic episodes with normal muscle tone, no loss of consciousness, and no abnormal movements in the eyes or extremities. Therefore, this was excluded because the patient was well during sleep despite having many attacks during the day, the symptoms were related to feeding, and her video electroencephalogram (EEG) was normal. Since detailed cardiac evaluation is required for an infant hospitalized with cyanosis and apnea, echocardiography was performed due to a murmur heard and revealed a 2–3 mm patent foramen ovale. There was no structural anomaly suggestive of aortic arch anomaly or cyanotic heart disease. After exclusion of cardiac anomaly and epilepsy possibilities, empirical antireflux treatment was initiated with a diagnosis of GER disease.

**Fig. 1:**
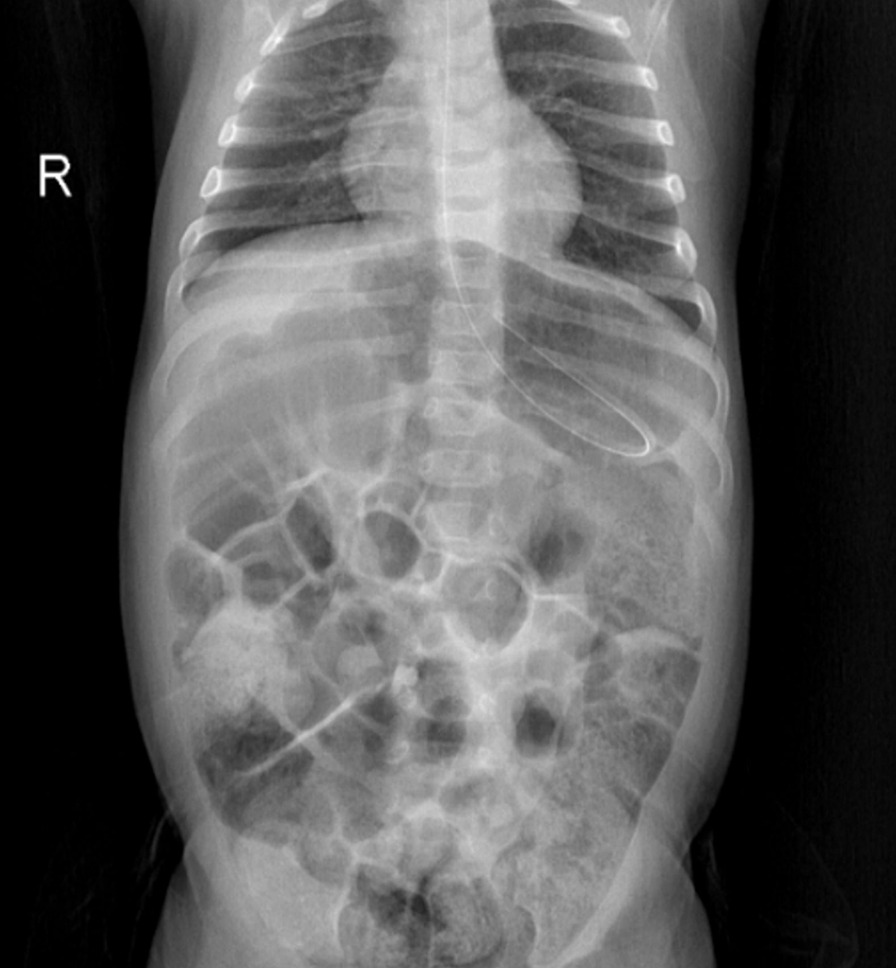
Excessive gas shadows in intestines and abnormally located fundus gas on the abdominal radiograph

**Fig. 2:**
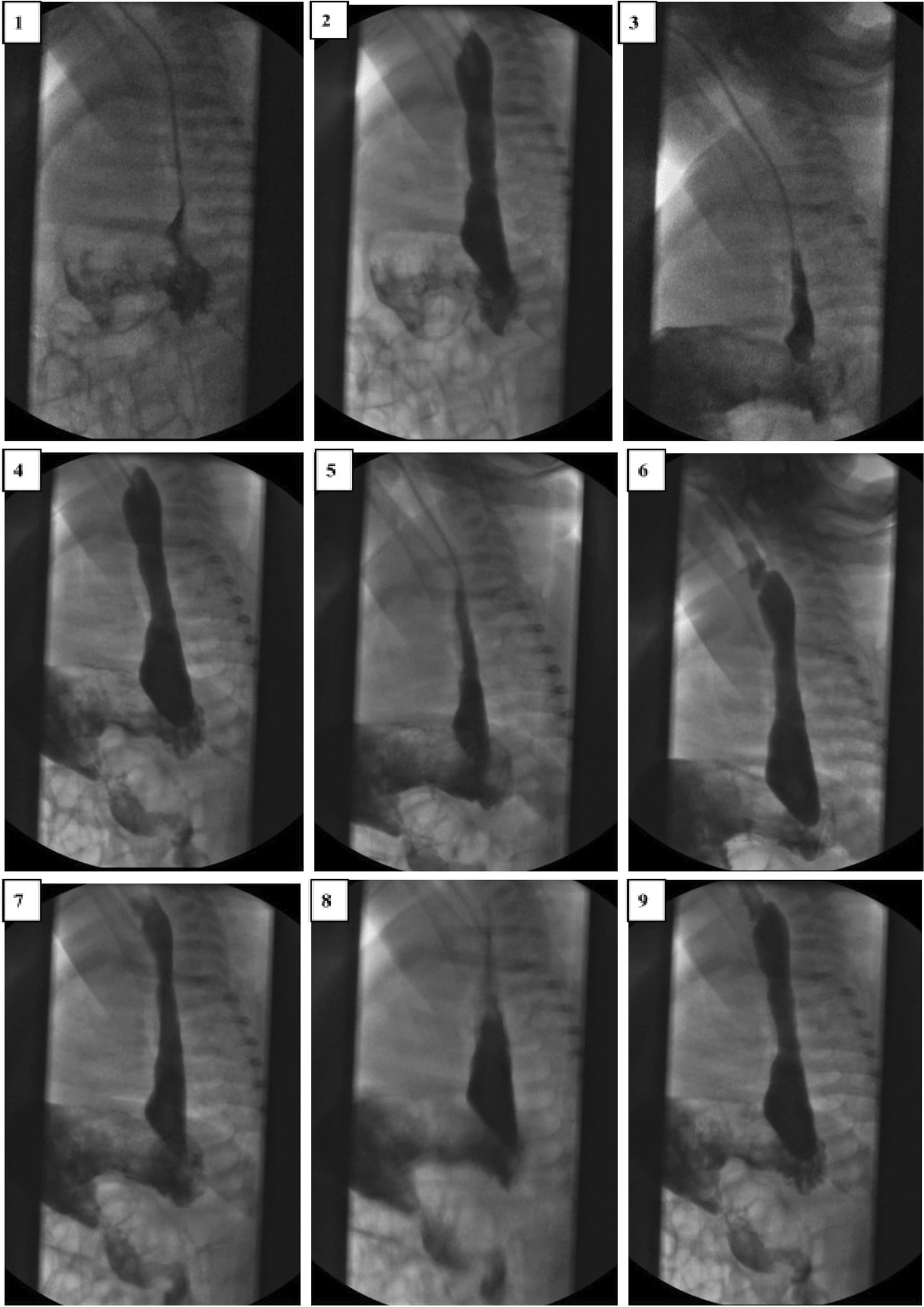
Reflux repeats several times and up to the proximal part of the esophagus on the double-contrast esophagus–stomach–duodenum

**Fig. 3:**
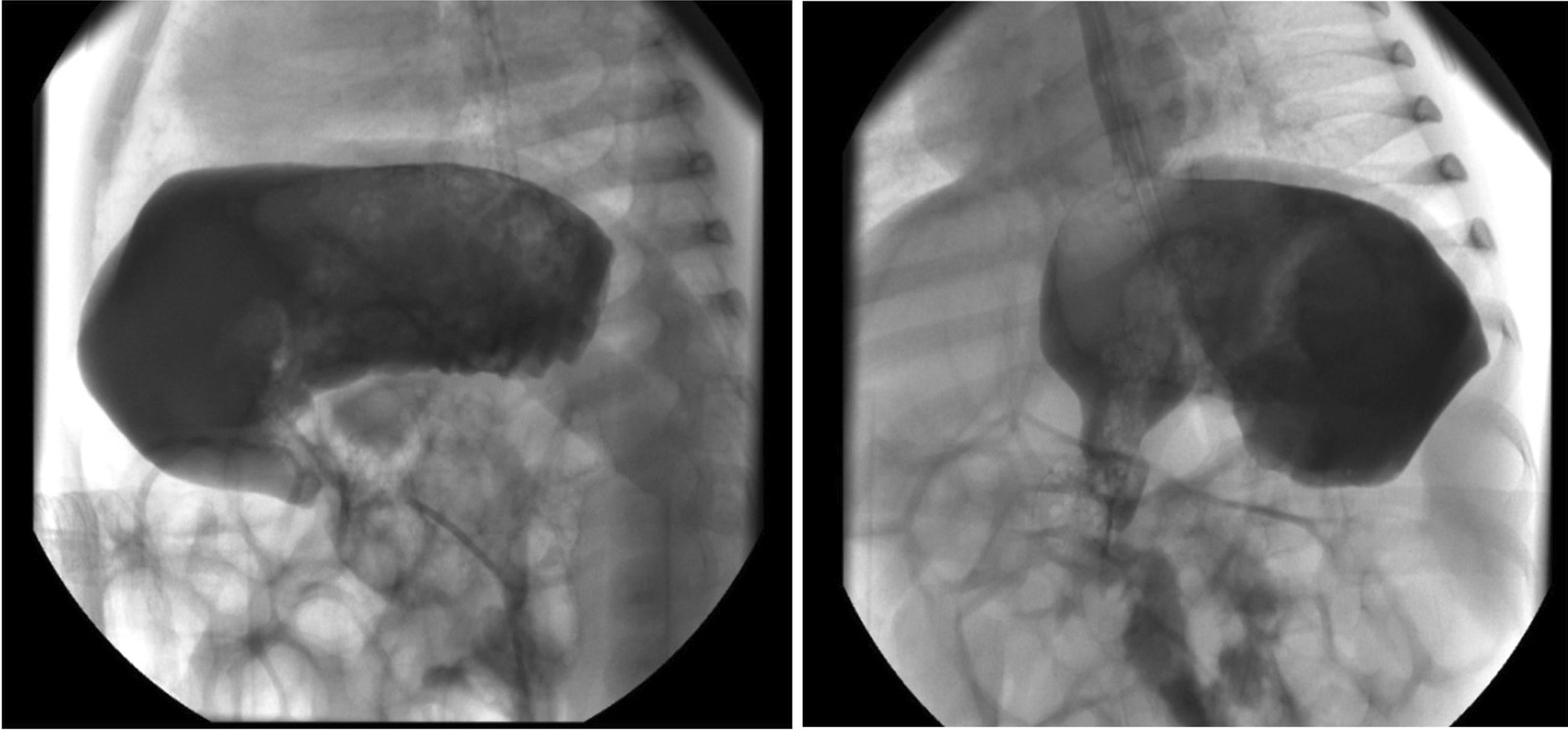
Gastric organo-axial malrotation

Oral intake was discontinued by inserting a nasogastric tube for tube feeding. On the abdominal radiograph, excessive intestinal gas shadows with an abnormal image of the fundus gas were visualized (Fig. 1). Double-contrast barium radiography was performed to determine anatomic abnormalities such as hiatal hernia, malrotation, or strictures and to evaluate for other conditions that might mimic or predispose to GERD due to the patient having alarm signs. The orally administered contrast freely passed through the esophagus into the stomach, and the oral and pharyngeal phases of swallowing were natural. Esophageal contours, peristaltic activity, and passage were normal without tracheoesophageal fistula. During the esophagram, severe reflux was observed that repeated at least three times up to the proximal part of the esophagus (Fig. 2). Also, there was an upward rotation of the stomach along its long axis, which resulted in displacement of the greater curvature adjacent to the abdominal face of the diaphragm and inversion of the greater curvature above the lesser curvature (Fig. 3).

The patient benefited from tube feeding when expressed breast milk was given through the nasogastric catheter at 40 ml per hour. It was observed that her cough regressed, and the apnea episodes disappeared. Since the patient had no reflux symptoms for a week and gained weight after 20 days of follow-up, she was discharged home with nasogastric tube feeding during home follow-up. She will undergo an elective surgical procedure when her weight and age are appropriate with the aim of preventing gastric malrotation from progressing to volvulus or intestinal ischemia.

## Discussion and conclusion

Regurgitation, defined as passage of refluxed contents into the pharynx or mouth, is a normal physiologic process occurring several times a day in healthy infants. Prevalence peaks at the age of 2–4 months then decreases, with only 5% of infants still regurgitating daily at 18 months [7]. Although regurgitation is a characteristic symptom of reflux in infants, it is not sufficient for the diagnosis of GER disease. On the other hand, there is a continuity between physiologic reflux and GER disease, leading to different manifestations and complications depending on the individual’s sensitivity, defense mechanisms, and possible anatomical variation [2].

The pathophysiology of GER disease is multifactorial, but the primary mechanism is transient relaxation of the lower esophageal sphincter where gastric contents enter the esophagus. Certain conditions and underlying diseases predispose to severe gastroesophageal reflux, including neurologic impairment, congenital anatomical abnormalities, and chronic pulmonary disease such as cystic fibrosis [2, 8]. Gastric malrotation is one of the anatomical conditions that may cause severe reflux and life-threatening complications. Organo-axial type of malrotation occurs when the stomach rotates along its long axis and becomes obstructed, with the greater curvature being displaced superiorly and the lesser curvature located more caudally in the abdomen [9]. It is very rare in the pediatric population and presents with symptoms including abdominal pain, recurrent vomiting, hematemesis, gastric distension, malnutrition, and failure to thrive. Senocak et al. reported 21 patients who underwent surgical intervention with the diagnosis of chronic gastric malrotation. On admission, the common symptoms were vomiting since birth and failure to thrive in all patients [10]. Similarly, Samuel M et al. reported ten children diagnosed with gastric malrotation-associated GER with frequent symptoms such as colicky abdominal pain before nonbilious vomiting, upper abdominal distension, hematemesis, and failure to thrive [11]. Besides, respiratory symptoms such as cough, apnea, and recurrent pneumonia could be observed in patients with gastric malrotation. Kose et al. reported 14 patients admitted primarily to the pulmonology department with respiratory symptoms and evaluated for underlying conditions of recurrent respiratory symptoms, such as cystic fibrosis and immune deficiency. However, all patients had gastric malrotation without abdominal symptoms. They observed that the only extrapulmonary manifestation was failure to thrive, and only two patients had apnea, which was seen in our case as the primary symptom [4]. Data are insufficient concerning the respiratory symptoms directly associated with gastric malrotation. Malrotation associated with occult GER could probably be responsible for recurrent respiratory problems, and further prospective observations are needed to demonstrate the respiratory manifestations.

Especially infants with gastric malrotation have been thought to be at risk for GER disease. These infants usually have a defective gastroesophageal junction, which may result from the same conditions that lead to the interference of normal intestinal rotation during fetal development [12]. Also, signs of GER cannot be easily interpreted and distinguished from some conditions such as sleep disturbances, pertussis-like syndrome, cow milk allergy, or BRUE because they cannot verbalize their symptoms. BRUE is a potential differential diagnosis for infants presented to the emergency department with reported history of stopping breathing, choking, and turning blue at home. It should be clear that BRUE is diagnosed only when there is no other explanation for a qualifying event, following an appropriate history and thorough physical examination. On the other hand, GER is common in infants presenting with BRUE and thought to be directly associated with reflex hypoxemic events [13]. Although reflux causes physiologic apnea, it causes pathologic apneic episodes in only a small number of newborns and infants. Wenzl et al. investigated infants with respiratory symptoms simultaneously with multichannel intraluminal impedance, pH monitoring, and polysomnography. They concluded that there is a significant correlation between apneic episodes and GER [14].

In general, the urgency and severity of symptoms dictate management. Acute gastric malrotation is a true surgical emergency because it could be life-threatening if not treated quickly. Obstructive symptoms, including sudden onset of persistent, nonbilious emesis, frequently accompanied by epigastric pain and distention, make it easier to consider the diagnosis as acute malrotation. On the other hand, many patients have a less severe or incomplete rotation of less than 180°, as in our case. These patients usually lack clinical symptoms of obstruction and present with a more chronic and atypical clinical course. The underlying anatomical abnormality may be overlooked, and symptoms such as failure to thrive, intermittent vomiting, and respiratory distress are more likely to be attributed to GER disease. Even though complaints of undiagnosed chronic gastric malrotation may be resolved with antireflux therapy, the partial gastric malrotation can predispose to future volvulus. However, chronic malrotation can be treated conservatively unless the symptoms are severe, and it is unclear whether asymptomatic patients should be treated or followed up clinically [3, 15].

In infants with GER, the main roles of history and physical examination are ruling out other disorders that may present with vomiting and identifying complications. Alarm symptoms such as apnea, hematemesis, excessive irritability, or failure to thrive merit a broader differential diagnosis and additional investigation than GER disease alone. Also, atypical presentations, complicated GER, or failure to respond to empiric management are indications for further diagnostic evaluations. Imaging studies provide some utility to evaluate infants and children with symptoms that are not responsive to conventional therapies and determine anatomical abnormalities and other conditions that may cause GER disease. Abdominal radiographs in the patients with acute fulminant gastric malrotation reveal findings highly suggestive of the diagnosis. Also, in the case of partial malrotation, inversion of the greater curvature relative to the lesser curvature and a horizontally oriented fundus gas suggest the organo-axial type of configuration. Although findings on abdominal radiographs can be highly suggestive of the diagnosis of gastric malrotation, upper gastrointestinal series remains the diagnostic study of choice. Computer tomography can define gastric malrotation and exclude other abdominal pathology as the source of symptoms but is not indicated in the initial evaluation. Also, endoscopic decompression may be attempted for a patient presenting with acute fulminant gastric malrotation after the initial intervention [16].

In conclusion, GER disease guidelines recommend careful evaluation of differential diagnoses when symptoms are nonspecific and life-threatening. If complaints persist despite adequate medical treatment, careful attention should be given to the presence of alarm signs that may suggest underlying conditions presenting with GER. Gastric malrotation is more common than generally thought and should be specifically looked for in young children with reflux accompanied by respiratory symptoms. Although imaging studies are not enough to establish the diagnosis of GER disease in infants, they carry some utility to evaluate children with symptoms that are not responsive to conventional therapies. Pediatric population-based studies of reflux symptoms are insufficient and are a priority for further research.

## Data Availability

All data generated or analyzed during this study are included in this published article.

## Abbreviations

GER: Gastroesophageal reflux
BRUE: Brief resolved unexplained events
PCR: Polymerase chain reaction
EEG: Electroencephalogram
